# Time-Series Forecasting of PM_2.5_ and PM_10_ Concentrations Based on the Integration of Surveillance Images

**DOI:** 10.3390/s25010095

**Published:** 2024-12-27

**Authors:** Yong Wu, Xiaochu Wang, Meizhen Wang, Xuejun Liu, Sifeng Zhu

**Affiliations:** 1 School of Geographical Sciences, Fujian Normal University, Fuzhou 350117, China; wuyong3216@fjnu.edu.cn; 2 Shanghai Surveying and Mapping Institute, Shanghai 200063, China; 3 School of Geography, Nanjing Normal University, Nanjing 210023, China; wangmeizhen@njnu.edu.cn (M.W.); liuxuejun@njnu.edu.cn (X.L.); 4 Shanghai Institute of Satellite Engineering, Shanghai 201109, China

**Keywords:** PM_2.5_ and PM_10_ forecasting, deep learning, LSTM, VGG16-LSTM, multi-source data fusion, surveillance images

## Abstract

Accurate and timely air quality forecasting is crucial for mitigating pollution-related hazards and protecting public health. Recently, there has been a growing interest in integrating visual data for air quality prediction. However, some limitations remain in existing literature, such as their focus on coarse-grained classification, single-moment estimation, or reliance on indirect and unintuitive information from visual images. Here we present a dual-channel deep learning model, integrating surveillance images and multi-source numerical data for air quality forecasting. Our model, which combines a single-channel hybrid network consisting of VGG16 and LSTM (named VGG16-LSTM) with a single-channel Long Short-Term Memory (LSTM) network, efficiently captures detailed spatiotemporal features from surveillance image sequences and temporal features from atmospheric, meteorological, and temporal data, enabling accurate time-series forecasting of PM_2.5_ and PM_10_ concentrations. Experiments conducted on the 2021 Shanghai dataset demonstrate that the proposed model significantly outperforms traditional machine learning methods in terms of accuracy and robustness for time-series forecasting, achieving *R*^2^ values of 0.9459 and 0.9045 and RMSE values of 4.79 μg/m^3^ and 11.51 μg/m^3^ for PM_2.5_ and PM_10_, respectively. Furthermore, validation results on the datasets from two stations in Kaohsiung, Taiwan, with average *R*^2^ values of 0.9728 and 0.9365 and average RMSE values of 1.89 μg/m^3^ and 5.69 μg/m^3^ for PM_2.5_ and PM_10_ using a pretrain–finetune training strategy, confirm the model’s adaptability across diverse geographical contexts. These findings highlight the potential of integrating surveillance images to enhance air quality prediction, offering an effective supplement to ground-level environmental monitoring. Future work will focus on expanding datasets and optimizing network architectures to further improve forecasting accuracy and computational efficiency, enhancing the model’s scalability for broader regional air quality management.

## 1. Introduction

Air pollution is widely recognized as a major global environmental health threat, with prolonged exposure linked to significant impacts on human health and life expectancy. According to the World Health Organization, ambient air pollution is responsible for millions of premature deaths annually [1], underscoring the urgent need for effective air quality forecasting. Accurate forecasts enable early warnings, which are essential for mitigating health risks and improving overall quality of life.

Over the years, researchers have developed various air quality forecasting methods, broadly categorized into numerical simulation methods and statistical methods. Numerical simulation models, such as the Community Multiscale Air Quality (CMAQ) model [2,3,4,5], the Nested Air Quality Prediction Modeling System (NAQPMS) [6], and the Weather Research and Forecasting model coupled with chemistry (WRF-Chem) [7,8], are grounded in meteorological principles and simulate pollutant emission, dispersion, and transformation processes through complex physical and chemical mechanisms [9]. While theoretically robust and interpretable, these models are computationally intensive due to their reliance on extensive high-quality input data with fine-grained spatial and temporal resolutions and intricate meteorological and chemical coupling processes. These constraints often restrict their scalability and practicality for real-time urban monitoring applications [10,11,12,13,14].

To overcome the limitations of physical simulation, statistical methods are frequently employed for air quality forecasting. These methods leverage historical data, including meteorological variables, pollutant concentrations, and temporal trends, to statistically infer future atmospheric conditions. Widely used techniques include Multiple Linear Regression (MLR) [15], Auto Regressive Moving Average (ARMA) [16], Random Forest (RF) [17], Support Vector Machines (SVM) [18], Artificial Neural Networks (ANN) [19], and various hybrid approaches [20,21]. Among these, Recurrent Neural Networks (RNN) and Long Short-Term Memory (LSTM) networks [22,23] have gained attention due to their ability to learn from time-series data and capture dynamic pollutant trends, often achieving promising forecasting performance.

Both traditional physical and statistical models primarily rely on multi-source numerical data, such as pollutant concentrations and meteorological observations. As advancements in computer vision and deep learning, visual data, which can reflect light transmission and scattering properties [24,25], has become a valuable resource for atmospheric inversions. This has driven researchers to explore the integration of visual imagery with numerical data to provide more holistic and accurate forecasts. For instance, Xia et al. [26] proposes a multi-modal deep-learning model to integrate high spatial resolution remote-sensing images and time-series air quality data, improving future PM_2.5_ forecasts of multiple stations. Rowley and Karakus [27] designed a multimodal AI network named AQNet to predict air pollutants of NO_2_, O_3_, and PM_10_ by combining multi-spectral Sentinel-2 satellite imagery, low-resolution tropospheric NO_2_ concentration data from Sentinel-5P satellite, and tabular ground measurement data. These studies demonstrate the promise of visual data integration. However, satellite remote sensing data, despite its broader spatial coverage, often suffers from temporal delays and reduced real-time applicability due to the inherent limitations of data acquisition and processing.

Surveillance imagery presents a compelling alternative to satellite data, offering continuous and high-temporal resolution monitoring in localized settings. With widespread deployment in urban environments, surveillance cameras can capture dynamic environmental changes in real time. Studies [28,29,30,31] have statistically analyzed various image features such as color, edge, and texture under different air pollution conditions, demonstrating the potential of surveillance images for pollutant estimation. Surveillance-image-based air quality monitoring provides an innovative, real-time, and ground-level measurement technology for air quality management. It can serve as a valuable supplement to traditional ground-based air quality monitoring stations for enhancing the spatial and temporal resolution of environmental observations, further supporting decision-making for pollution control and public health management. However, existing research leveraging surveillance images for air quality prediction remains limited. Most studies focus on the coarse-grained classification of pollutant levels [32,33,34,35] or single-moment pollutant estimation [36,37,38,39] rather than time-series forecasting. Additionally, many rely on indirect information, such as traffic density [40], which is challenging to extract accurately from images captured under severe pollution. These limitations highlight the need for a direct and fine-grained prediction approach that fully exploits the potential of surveillance images for quantitative time-series forecasting.

To address these gaps, this study proposes a dual-channel integrated deep learning model that concurrently processes multi-source numerical data and surveillance images to forecast multi-time PM_2.5_ and PM_10_ concentrations. Specifically, a VGG16-LSTM single-channel network is employed to directly extract detailed spatiotemporal visual features from surveillance image sequences, while an LSTM single-channel network captures temporal features from time-series numerical data. By fusing these two distinct features, the dual-channel integrated model is endowed with the capability to handle multi-source heterogeneous data. To validate the effectiveness of the proposed model, experiments were conducted using the 2021 Shanghai dataset and Kaohsiung dataset, comparing it with several commonly used statistical models, along with detailed evaluation experiments. The main contributions of this work are summarized as follows:

- We develop a dual-channel deep learning network architecture, enabling the efficient integration of surveillance images and numerical data for accurate multi-time quantitative forecasting;
- The proposed model improves the PM_2.5_ and PM_10_ forecasting accuracy by capturing detailed spatiotemporal features from surveillance images, compared to commonly used statistical models;
- Performance evaluation validates the model’s robustness for long time-series forecasting and its transferability across geographically diverse datasets.

The remainder of this paper is organized as follows: Section 2 details the dataset, data preprocessing methods, and the proposed dual-channel integrated model. Section 3 presents the experimental results, including performance evaluation, comparison with existing methods, and transferability analysis, and discusses the study’s limitations and potential implications. Finally, Section 4 concludes the paper with a summary of findings and directions for future research.

## 2. Materials and Methods

### 2.1. Dataset

**Figure 1:**
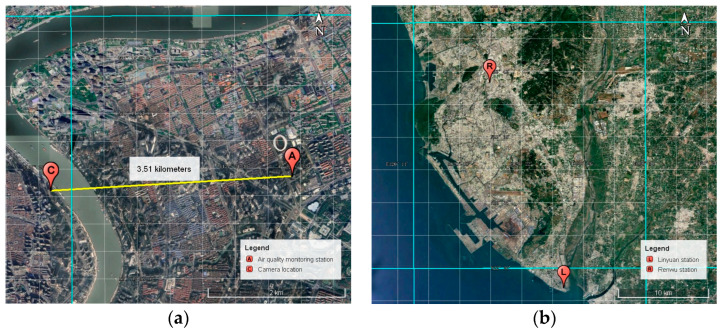
Data acquisition location maps. (**a**) Shanghai dataset: the distance between the surveillance camera location and the air quality monitoring station is 3.51 km, and (**b**) Kaohsiung dataset: each surveillance camera location and the corresponding air quality monitoring station are co-located at the same site, where the cyan grid represents meteorological data units.

It is well established that air quality dynamics are influenced by multiple factors, including interactions among pollutants, coupling between successive processes, and effects from meteorological and other environmental conditions [41,42]. Numerous studies [43,44,45] have confirmed the value of incorporating data related to these influencing factors as inputs in air quality predictions. Building on this foundation and considering data accessibility, we incorporate routinely monitored indicators from atmospheric, meteorological, and temporal factors as supplementary inputs, alongside surveillance images for PM_2.5_ and PM_10_ concentration forecasting. Following previous research, raw data were collected from various sources associated with the monitoring station in Pudong New Area (Station ID: 1149A, located at 121.533° E, 31.2284° N). The data acquisition locations are shown in Figure 1a. A comprehensive multi-source dataset was compiled, including single-camera images, six conventional air pollutant indicators, seven common meteorological variables, and two temporal parameters, as summarized in Table 1.

Specifically, the temporal factors utilized in this study refer to the month and hour parameters, which were directly extracted from the metadata of other collected data, eliminating the need for separate collection. The other data sources are as follows:

- Image Data: Camera images were acquired via web scraping from the official website of the Shanghai Municipal Bureau of Ecology and Environment (https://sthj.sh.gov.cn/). A total of 8499 surveillance images were collected, captured hourly from 1 January 2021 to 31 December 2021, with a resolution of 584 × 389 pixels.
- Atmospheric Data: The detailed measurement of various air pollutants from all stations nationwide can be obtained from the China National Environmental Monitoring Center (http://www.cnemc.cn/). In this study, 8374 valid hourly concentration records of six conventional air pollutants—PM_2.5_, PM_10_, SO_2_, NO_2_, O_3_, and CO—were downloaded for the entire year of 2021 from the monitoring station in Pudong New Area.
- Meteorological Data: Based on research related to the CHAP dataset [46,47], seven meteorological variables were selected as air quality forecasting factors: precipitation (PRE), surface pressure (SP), temperature (TEM), evaporation (ET), relative humidity (RH), wind speed (WS), and wind direction (WD). These hourly meteorological values were primarily collected from the fifth-generation reanalysis data (ERA5, https://cds.climate.copernicus.eu/) (accessed on 6 September 2022) provided by the Copernicus Climate Change Service (C3S), with a spatial resolution of 0.25° × 0.25°.

Missing data and outliers were inevitable due to instrument failures or extreme weather conditions. In our dataset, meteorological and temporal data records were complete, whereas the image data had only a few missing instances, and atmospheric data exhibited a small number of anomalous negative values and missing entries. These anomalies and missing data were uniformly treated as missing values, resulting in a total of 8126 complete and valid data records across the dataset. To generate a sufficient number of samples for model training, missing data were interpolated using different methods depending on the length of the gap and the data type: for pollutant data with gaps of 4 h or less, the average of the two hours before and after the gap was used, and for longer gaps, the average of the corresponding time points on the previous and subsequent days was used [48]; for image data with gaps of 4 h or less, images from the previous or subsequent two hours were used, and no interpolation was performed for longer gaps. The interpolation increased the number of complete hourly records from 8126 to 8583. Subsequently, a sliding window approach [49] was employed to generate sequence samples for model training and testing, producing 8365 sequence samples for a 24-h forecasting horizon with 12-h historical data and 12-h forecasted data.

### 2.2. Dual-Channel Integrated Network

Given the interactions among air pollutants, the influence of meteorological conditions, the temporal dependence of air pollution variation, and visual changes associated with particulate matter fluctuations, this study utilizes multi-source heterogeneous data, including historical pollutant records, meteorological variables, time information, and visual images to forecast future PM_2.5_ and PM_10_ trends. Hourly air pollutant, meteorological, and temporal data can be processed as single numerical values, allowing seamless integration into a unified model. However, the image at each time point is represented as a multidimensional array, which differs significantly in structure from numerical data. This structural disparity complicates feature extraction, as a single model may struggle to effectively process both data types without sacrificing critical details. Reducing image data to a single value, for example, would cause substantial information loss. To address this, we designed a dual-channel integrated network framework that independently extracts and processes features from each data type, preserving the unique characteristics of both for improved predictive accuracy.

LSTM-based deep learning sequence models are widely applied in existing studies to forecast future air quality based on historical time-series data [50,51]. Through the gate mechanism, the LSTM model progressively transforms features into a new feature space that captures rich temporal information, facilitating more accurate forecasts. In this study, an LSTM network is similarly implemented as a branch of the dual-channel network to extract the temporal features from numerical data, including pollutant concentrations, meteorological conditions, and time information. The LSTM single-channel network comprises a single LSTM layer with 200 hidden neurons, and its output is then prepared for the next stage of integrated learning.

Building on the successful application of the VGG16-LSTM model for image-based air quality estimation in our previous work [36], where the VGG16 component extracts spatial features from individual surveillance images and the LSTM layer captures temporal correlations in image sequences, linking visual changes with particulate matter fluctuations over time; this study continues to use its feature extraction layers as the second branch of our designed network. In the VGG16-LSTM single-channel network, we modified the VGG16 architecture by removing its fully connected and output layers, retaining only the feature extraction layers to capture spatial features. These features are then passed to an LSTM layer with 512 neurons, which captures temporal dependencies between image sequences, generating deep and abstract spatiotemporal features.

To fully exploit the advantages of multi-source heterogeneous data, we fuse the temporal features extracted by the LSTM single-channel network with the spatiotemporal features extracted by the VGG16-LSTM single-channel network at the feature level. This fusion integrates surveillance image data with traditional forecasting factors, providing a holistic approach to PM_2.5_ and PM_10_ forecasting. Following feature fusion, two fully connected layers are applied to map the learned features to the output. The first fully connected layer, containing 200 neuron nodes, further refines the fused features into high-level abstract representations. The second fully connected layer serves as the output layer, designed to meet the goal of this study—multi-time forecasting of PM_2.5_ and PM_10_ concentrations. The output layer generates a two-dimensional array, where the dimensions correspond to the forecasting horizon and the number of forecasted variables. The number of neurons in the output layer matches the forecasting horizon, and an activation function is applied to model the nonlinear regression relationship between the input features and the forecasted targets.

**Figure 2:**
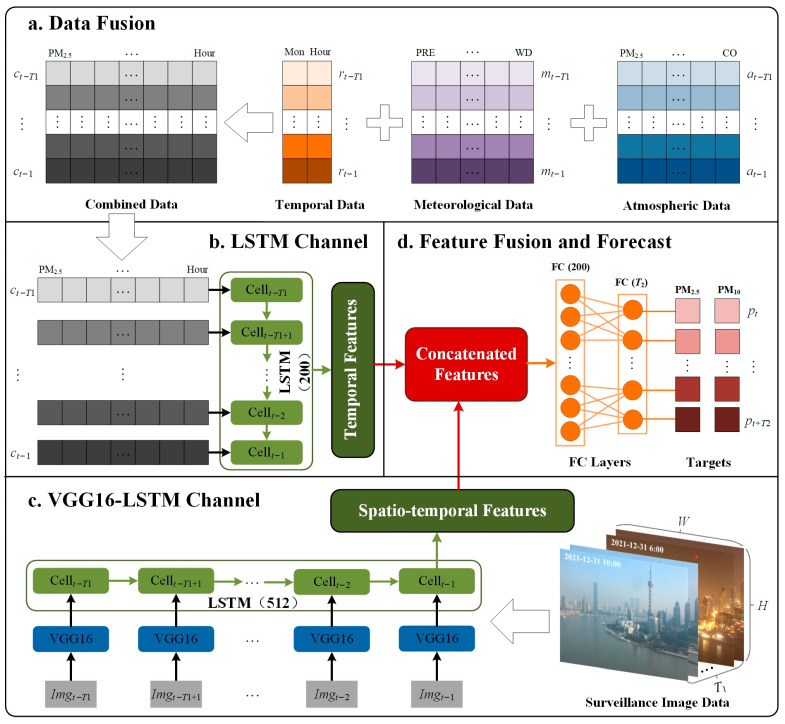
The network architecture of dual-channel integrated model. (**a**) Data fusion module: Combines multi-source homogeneous numerical data into a unified format for processing; (**b**) LSTM single-channel network module: Extracts temporal features from atmospheric, meteorological, and temporal data; (**c**) VGG16-LSTM single-channel network module: Extracts spatiotemporal features from surveillance image sequences; and (**d**) Feature fusion and forecasting module: Merges features and outputs time-series predictions of PM_2.5_ and PM_10_ concentrations.

In summary, the proposed PM_2.5_ and PM_10_ forecasting model consists of four key modules, as illustrated in Figure 2. The first is the data fusion module, which merges homogeneous numerical data from different sources into a two-dimensional array with the shape (T_1_, 15) for supervised learning. The second module is the LSTM single-channel network, which is responsible for extracting temporal features from the homogeneous numerical data and producing an output feature vector of length 200. The third module is the VGG16-LSTM single-channel network, which extracts spatiotemporal features from surveillance image data, formatted as (T_1_, H, W, 3), generating an output feature vector of length 512. The final module is the feature fusion and forecasting module, which combines the outputs from the two single-channel networks. After fusion, the combined feature vector has a length of 712, and the final model output is produced through two fully connected layers, resulting in an output size of T_2_ × 2. Here, T_1_ represents the time horizon of historical data used for forecasting, T_2_ refers to the future time period for being predicted, and H and W are the height and width of input images, respectively.

The dual-channel deep learning network builds upon the existing LSTM and VGG16-LSTM architectures but distinguishes itself by utilizing two branch networks to extract detailed features from multi-source numerical data and surveillance images, effectively preserving the unique characteristics of heterogeneous data. Its multi-step, multi-output design enables long-term quantitative forecasting of PM_2.5_ and PM_10_ concentrations, overcoming the limitations of single-moment and coarse-grained estimations. By leveraging the complementary strengths of numerical and visual data, this integrated approach aims to improve forecasting accuracy and demonstrate the potential of surveillance-image-assisted methodologies in advancing air quality measurement.

## 3. Results and Discussion

### 3.1. Model Implementation

The models in this study were implemented using Python 3.7 and the TensorFlow deep learning framework on a server configured with an Intel Xeon Silver 4216 CPU @ 2.10 GHz, an NVIDIA GeForce GTX 2080Ti GPU, and a Windows 10 operating system. Key parameters were carefully initialized to optimize model performance. Similar to the model setup of VGG16-LSTM, mean squared error (MSE) was selected as the loss function to quantify training accuracy, while a learning rate of 0.00001 was chosen to balance model weight updates. A batch size of 8 was used to maintain computational efficiency without overloading memory. To ensure compatibility with the input of VGG16, all images were resized to 224 × 224 pixels, and their pixel values were scaled from a 0–255 range to a 0–1 range to accelerate convergence. The values for pollutant concentrations and meteorological variables were normalized to a 0–1 range based on their respective upper limits (e.g., PM_2.5_ concentration was capped at 500 [52]).

Two-fold cross-validation was employed to robustly assess model performance. The dataset was split into two equal parts, with each half alternately serving as the training and testing sets. The average performance across these folds was considered the final prediction result. The proposed model’s predictive accuracy was evaluated using two widely recognized regression metrics: the coefficient of determination (*R*^2^) and the root mean squared error (RMSE). The *R*^2^ metric, ranging from 0 to 1, indicates how closely the predicted values align with the actual air quality measurements, while RMSE quantifies the average prediction error. A higher *R*^2^ and a lower RMSE reflect superior model performance.

### 3.2. Overall Accuracy

**Figure 3:**
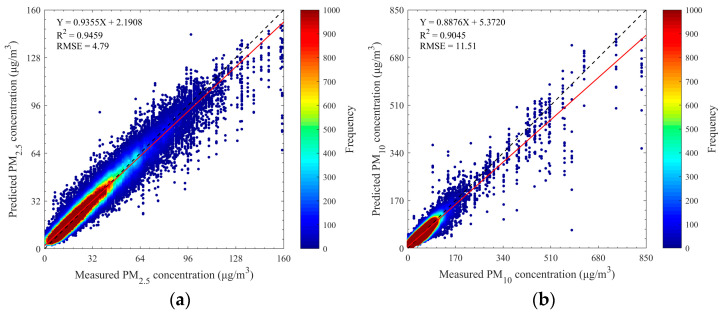
Scatter density plots of forecast results of two atmospheric particulate matters. (**a**) PM_2.5_ and (**b**) PM_10_. Black dashed lines denote 1:1 lines, and red solid lines denote best-fit lines from the linear regression. Note: A single ground truth value (*x*-axis) may correspond to multiple predicted values (*y*-axis) in the scatter plot because each ground truth value can appear at different forecasted time points in different sample sequences when using the sliding window approach for sample generation.

To validate the forecasting performance of the proposed method, the dual-channel integrated model was tested on the pre-processed experimental dataset. The experimental setup is consistent with the description in Section 3.1. Figure 3a,b present scatter density plots of the 12-h forecast results versus actual measured values for PM_2.5_ and PM_10_ concentrations. Statistical analysis reveals high *R*^2^ values and low RMSE for two pollutants: 0.9459 and 4.79 µg/m^3^ for PM_2.5_, 0.9045 and 11.51 µg/m^3^ for PM_10_. Additionally, the scatter points align closely around the 1:1 line with high sample density and steep fitting slopes (approximately 0.9355 and 0.8876), indicating strong consistency between the predicted and observed values. This confirms the feasibility and effectiveness of the dual-channel integrated deep learning model for PM_2.5_ and PM_10_ quantitative forecasting.

However, further examination of Figure 3 shows an underestimation of pollutant levels in high-value regions. This underestimation primarily stems from the uneven distribution of samples, with fewer high-concentration samples available for training, leading to weaker learning capability for high-concentration samples and increased prediction errors. Despite this, the multi-source data fusion-based dual-channel integrated model demonstrates excellent overall performance, achieving high accuracy for PM_2.5_ and PM_10_ forecasting.

### 3.3. Performance Analysis

#### 3.3.1. Comparison with Other Methods

Additionally, the proposed dual-channel integrated model was compared with widely used traditional statistical methods, including MLR, RF, and SVR, as well as the LSTM model. In the RF algorithm, the number of decision trees is a critical parameter influencing the model, and 1000 decision trees were set referring to Doreswamy et al. [53]. Following García Nieto et al. [18], a polynomial kernel function was selected for the SVR algorithm. The LSTM model used here corresponds to the LSTM single-channel network constructed in Section 2.2 and incorporates the same fully connected layer and output layer as the proposed dual-channel integrated model. To ensure a fair comparison, all methods were trained and tested on the same data splits of the processed Shanghai dataset, and all time-series numerical inputs were standardized to maintain consistency across models. Since models other than the dual-channel integrated model cannot directly process the multidimensional structure of surveillance images, we calculated the mean pixel value of each image. The averaged and normalized image data were then combined with other numerical data as inputs in these models to calculate 12-h time-series PM_2.5_ and PM_10_ concentrations as outputs. The forecasting performance was evaluated using metrics including *R*^2^ and RMSE for PM_2.5_ and PM_10_ concentrations. The forecasting results of different methods are presented in Table 2.

Among traditional machine learning algorithms, the RF algorithm achieved moderate forecasting performance, outperforming MLR and SVR in predicting both PM_2.5_ and PM_10_ levels. Compared to RF, the LSTM model significantly improved the forecast accuracy for PM_2.5_ and PM_10_ by 14.5% and 21.9%, respectively, highlighting the effectiveness of the sequence-based model for time-series forecasting. Notably, the dual-channel integrated model, which builds on the VGG16-LSTM network, achieved the highest prediction accuracy, with further improvements in *R*^2^ of 4.7% for PM_2.5_ and 6.6% for PM_10_. These results highlight the superiority of the VGG16-LSTM network, which enables the capture of detailed spatiotemporal features from surveillance images that traditional models and the single-channel LSTM approach might miss. The comparison also underscores the value of the dual-channel integrated model that preserves the unique characteristics of multi-source heterogeneous data for enhancing PM_2.5_ and PM_10_ quantitative forecasting performance.

#### 3.3.2. Influence of Different Forecasting Factors

**Figure 4:**
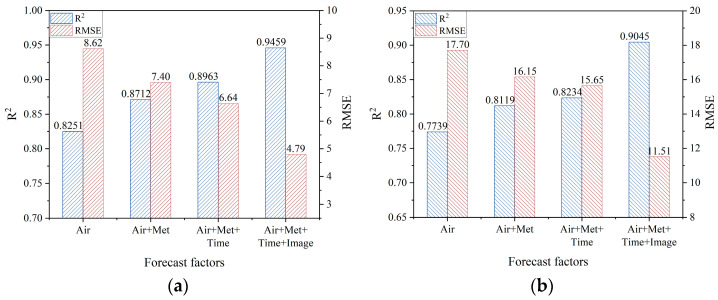
Comparison of prediction results on two atmospheric particulate matters with different combinations of forecast factors. (**a**) PM_2.5_ and (**b**) PM_10_.

To enhance forecasting accuracy, the study integrates various factors, including images, atmospheric data, meteorological data, and temporal data. Figure 4 compares the forecasting results obtained by using different combinations of these factors. The results based on combinations of atmospheric, meteorological, and temporal data (Air, Air + Met, Air + Met + Time) were obtained using the aforementioned LSTM model, while results using all data sources (Air + Met + Time + Image) were derived from the dual-channel model that integrates the LSTM and VGG16-LSTM networks.

The results indicate that the LSTM model combining atmospheric and meteorological data improves the *R*^2^ of PM_2.5_ and PM_10_ by 4% each while reducing RMSE by 1.22 µg/m^3^ and 1.55 µg/m^3^, respectively, compared to that relying solely on atmospheric data. Adding time information further increases *R*^2^ by an additional 2.5% and 1.2% and reduces RMSE by 0.76 µg/m^3^ and 0.5 µg/m^3^ for PM_2.5_ and PM_10_, respectively. Moreover, the dual-channel model, which integrates the VGG16-LSTM network to extract features from surveillance images, achieves an additional average *R*^2^ improvement of 6.5% for PM_2.5_ and PM_10_ forecasts.

Consistent with previous research, these findings demonstrate the effectiveness of the selected forecasting factors in improving air quality predictions. The inclusion of meteorological and temporal factors markedly enhances prediction accuracy, while the integration of image data, a heterogeneous source, further boosts the forecasting capability of PM_2.5_ and PM_10_.

#### 3.3.3. Performance on Different Forecast Time Lags

**Figure 5:**
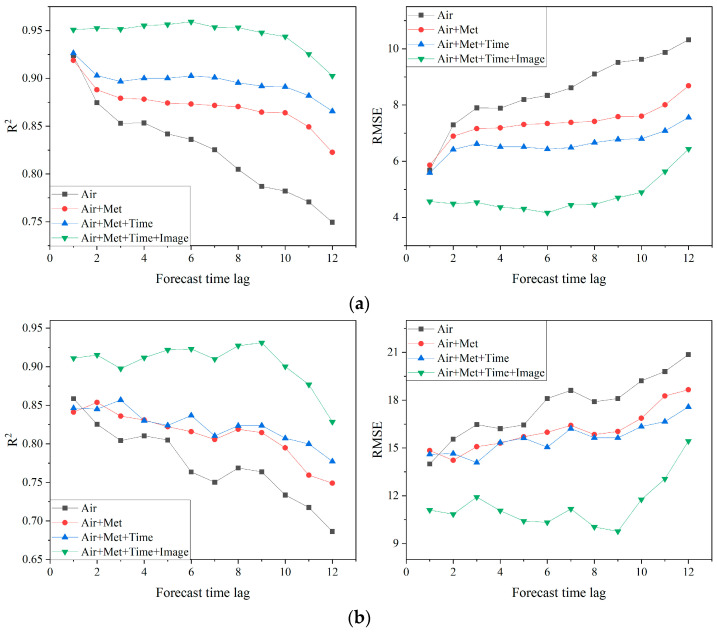
Comparison of forecast accuracy on two atmospheric particulate matters over different forecast time lags. (**a**) PM_2.5_ and (**b**) PM_10_.

The study evaluates the performance of the dual-channel integrated model against single-channel models over varying forecast time lags. The results, shown in Figure 5, indicate that the dual-channel model consistently achieves higher *R*^2^ values and lower RMSEs at each forecast hour compared to single-channel models. Even for 12-h forecasts, the dual-channel model maintains high accuracy, with *R*^2^ values exceeding 0.8 for PM_10_ and reaching as high as 0.9 for PM_2.5_. These findings further validate the superiority of the dual-channel model, which significantly outperforms single-channel models in terms of forecast performance.

Figure 5 also shows a general decline in forecast accuracy as the forecast time lag increases for both PM_2.5_ and PM_10_, a trend consistent with the decrease in autocorrelation of air pollutants over time [36,54], which impacts forecast effectiveness for longer horizons. Notably, the rate of decline in forecast performance is more pronounced for single-channel models, which is related to the number of forecasting factors involved. In contrast, the dual-channel model demonstrates more stable performance, particularly within the first 10 h, owing to its integration of multi-source data, including surveillance images. The analysis of forecast performance on different time lags underscores the effectiveness and robustness of the proposed dual-channel model on time-series forecasts.

#### 3.3.4. Performance Across Different Forecast Durations

**Figure 6:**
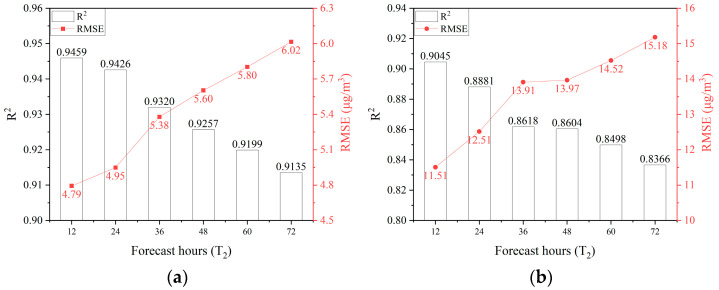
Comparison of prediction results on two atmospheric particulate matters across different forecast durations. (**a**) PM_2.5_ and (**b**) PM_10_.

Given the observed decline in forecast accuracy over longer time lags, it is critical to evaluate the feasibility of the dual-channel model for long-term air quality forecasting. To this end, additional experiments were conducted with forecast horizons of 24, 36, 48, 60, and 72 h while keeping the historical sequence length constant. The results are presented in Figure 6.

The dual-channel model demonstrates superior performance in extended time-series forecasts, maintaining high accuracy even when predicting future PM_2.5_ and PM_10_ concentrations up to 72 h ahead using only 12 h of historical data. Specifically, the model achieves *R*^2^ values of 0.91 and 0.84 for PM_2.5_ and PM_10_, respectively, with prediction errors of 6.02 µg/m^3^ and 15.18 µg/m^3^. These findings confirm the feasibility of applying the dual-channel deep learning model for long-term air quality forecasting.

However, a decline in *R*^2^ values and an increase in RMSE values are observed as the forecast horizon extends (including beyond 72 h), indicating an inverse relationship between forecast accuracy and duration. The longer the forecast duration, the greater the prediction uncertainty. Consequently, in practical applications, it is crucial to balance the trade-off between forecast accuracy and the desired forecast duration, ensuring the model meets the specific requirements of air quality management tasks.

#### 3.3.5. Influence of Data Interpolation

To enhance the continuity of time-series data and generate sufficient samples for model training, we applied interpolation to address missing records in the multi-source dataset. However, interpolating missing data may introduce additional uncertainty or errors in the predictions. To evaluate the impact of interpolation, we conducted a comparative experiment where missing data were either interpolated or removed entirely, and the resulting forecasting performance was analyzed.

After removing records with missing values, the dataset contained approximately 8126 complete records. Due to the discontinuity caused by missing data, the total number of 24-h sequence samples (T_1_ = 12, T_2_ = 12) generated by the sliding window method was significantly reduced to 5558. In contrast, interpolation increased the dataset to 8583 complete records, resulting in 8365 sequence samples—considerably improving the sample availability for training.

**Figure 7:**
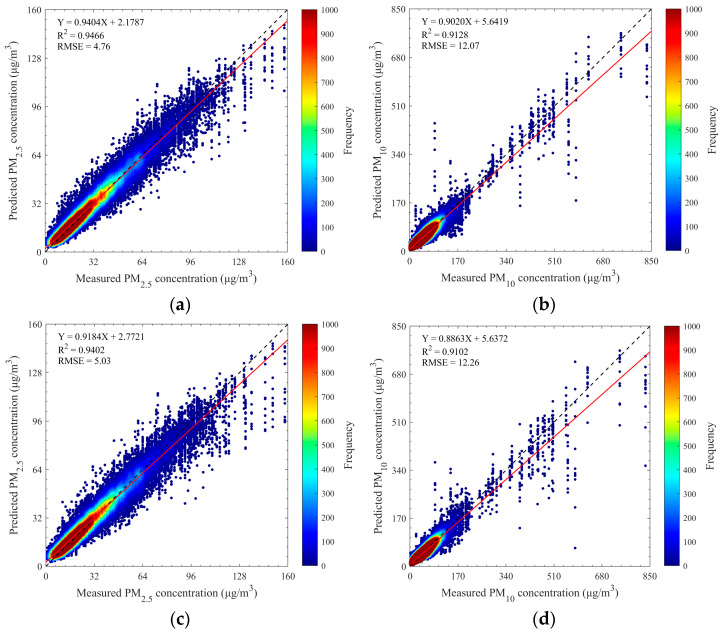
Scatter density plots of forecast results of two atmospheric particulate matter with different data processing approaches for missing values. (**a**) PM_2.5_ forecasts with data interpolation, (**b**) PM_10_ forecasts with data interpolation, (**c**) PM_2.5_ forecasts with data deletion, and (**d**) PM_10_ forecasts with data deletion. Black dashed lines denote 1:1 lines, and red solid lines denote best-fit lines from the linear regression.

The forecasting results for the two approaches, interpolation, and deletion, were compared using the 5558 identical sequence samples. Figure 7 presents the scatter density plots illustrating the comparison. Despite the reduced sample size when missing data were removed, our proposed method still achieved relatively high *R*^2^ values and low RMSE values for both PM_2.5_ and PM_10_ forecasts. Notably, the results on the interpolation-based dataset demonstrated slight improvements over that on the deletion-based dataset, likely benefiting from the increased sample size. It indicates that the interpolation method employed in this study is effective and does not introduce significant uncertainty or bias into the forecasts.

Although the sample size advantage was modest in this particular experiment, the importance of data volume in model training cannot be overstated. A larger sample size is crucial for improving model generalization and performance, particularly in long-term time-series forecasting. Therefore, interpolating missing data is not only beneficial but essential, and selecting an optimal interpolation method remains an important direction for future research.

#### 3.3.6. Transferability of the Model

In addition to the experiments conducted on the Shanghai dataset, the transferability of the proposed model was evaluated using datasets from two stations in Kaohsiung, Taiwan—Renwu (Station ID: 49, located at 120.332631° E, 22.689056° N) and Linyuan (Station ID: 52, located at 120.41175° E, 22.4795° N), whose locations are shown in Figure 1b. The image data provided by Hsieh et al. [55] and Kow et al. [56], with a resolution of 1280 × 720 pixels, were supplemented with air pollutant concentration data sourced from the Taiwan EPA website (https://data.moenv.gov.tw/) (accessed on 10 May 2022) and meteorological data from ERA5. After data preprocessing, 3462 and 3418 sequence samples were generated for the Renwu and Linyuan stations, respectively. Due to the spatial heterogeneity in air quality conditions and surveillance images between different locations, models trained on data from a fixed location often require retraining and adjustment when applied to data from other regions [57]. To assess adaptability, two approaches were used in this study: (1) Retrain: Retraining the dual-channel integrated model from scratch using the two Kaohsiung station datasets, and (2) Pretrain–finetune: Fine-tuning a pretrained model, initially trained on the Shanghai dataset, with the Kaohsiung data.

The predictive performance under both approaches is summarized in Table 3. The dual-channel integrated model demonstrated high accuracy in predicting air pollutant concentrations for both Kaohsiung stations under both training strategies, confirming the effectiveness and transferability of the proposed multi-source data integration method. Notably, the pretrain–finetuned model consistently outperformed the retrained model for all pollutants at both stations. Specifically, the *R*^2^ for PM_2.5_ increased by more than 0.05, and RMSE decreased by over 1.3 µg/m^3^; for PM_10_, the *R*^2^ improved by more than 0.04, and RMSE decreased by over 1.7 µg/m^3^.

The fine-tuning process enhanced the model’s predictive capacity by effectively leveraging the training data from both regions, thereby improving its adaptability. These findings validate the efficacy of the pretrain–finetune approach and highlight it as a recommended strategy for transferring models to new regions, particularly where local data are limited.

### 3.4. Limitations of This Study

While this study demonstrates significant advancements in air quality forecasting, several limitations must be acknowledged to provide a comprehensive understanding of its findings and implications. Firstly, the integration of surveillance images with atmospheric and meteorological data introduces challenges related to data availability and privacy. Meteorological and atmospheric pollutant records are generally reliable and accessible, but obtaining surveillance images can be hindered by legal, logistical, or policy constraints, resulting in a limited sample size and restricting broader generalization. Furthermore, the collected data often exhibit spatial discrepancies between different sources, as exemplified by the Shanghai dataset in this study. Specifically, the spatial distance between surveillance cameras and atmospheric or meteorological monitoring stations can lead to data misalignment, potentially introducing noise and reducing predictive accuracy. Despite these drawbacks, such datasets remain valuable and scarce resources for research. Future efforts should prioritize promoting open data sharing to create richer and more diverse datasets. This would enable more comprehensive assessments of the model’s generalizability across different regions and conditions while fostering deeper investigations into the effects of spatial inconsistencies and the development of advanced techniques to address and mitigate such discrepancies.

Secondly, the proposed dual-channel integrated model significantly improves forecasting accuracy by combining surveillance imagery with multi-source numerical data, but this comes at the cost of increased algorithmic complexity and resource demands. Specifically, the model’s parameter count increased approximately 75-fold, and the memory access cost (MAC) rose by nearly 2 million times, requiring up to 300 GB of computer memory to run the dual-channel integrated model. Such computational intensity necessitates high-performance GPUs and longer training durations, which may limit the scalability of this approach in resource-limited environments. Research on model optimization techniques, such as lightweight network architectures or model pruning, could help reduce computational overhead while maintaining accuracy.

Additionally, the resolution of surveillance images was resized to 224 × 224 pixels for model input in this study to balance computational efficiency and feature extraction. While sufficient for capturing general spatiotemporal patterns, this resolution may limit the model’s ability to detect fine-grained visual details that could enhance prediction accuracy. Higher-resolution images could potentially improve performance by capturing subtle atmospheric variations. Future studies should assess the trade-offs between image resolution, computational cost, and model accuracy to determine optimal configurations.

Finally, the use of interpolation methods to handle missing data inevitably introduces estimation errors, which can propagate through the model and affect prediction outcomes. Although the interpolation techniques employed in this study effectively increased data completeness and sample size, they may oversimplify the underlying temporal and spatial dynamics. More sophisticated approaches, such as machine learning-based imputation or dynamic temporal interpolation, should be explored to minimize biases and enhance data integrity.

## 4. Conclusions

To address the limitations of existing surveillance-image-based air quality prediction approaches, such as their focus on coarse-grained classification, single-moment estimation, or reliance on indirect and unintuitive information from surveillance images, this study develops a dual-channel deep learning model for time-series forecasting of quantitative air quality. This model efficiently integrates multi-source heterogeneous data while preserving their unique characteristics by leveraging a VGG16-LSTM single-channel network to directly extract spatiotemporal features from surveillance image sequences and an LSTM single-channel network to capture temporal features from atmospheric, meteorological, and temporal data. This design enables the model to achieve accurate multi-time PM_2.5_ and PM_10_ concentration forecasts, addressing the challenges of integrating complex, multimodal datasets.

The model’s performance was rigorously validated using the 2021 Shanghai dataset, demonstrating its effectiveness and feasibility. Compared to traditional air quality forecasting methods such as support vector regression and random forest, the dual-channel model significantly outperformed these baselines. The inclusion of surveillance image data not only enhanced forecast accuracy but also improved the robustness of the predictions, underscoring the value of integrating visual data into air quality forecasting. Furthermore, the model showed superior performance across various forecast durations, confirming its potential for long-term forecasting. To assess its adaptability, the model’s transferability was tested on datasets from two stations in Kaohsiung, Taiwan. Through both retraining and pretrain–finetuning approaches, the model demonstrated its ability to generalize across diverse geographical regions.

The proposed dual-channel integrated deep learning model achieved notable performance improvements, but we acknowledge certain limitations. Employing state-of-the-art deep learning architectures could further enhance computational efficiency and forecasting accuracy. Future work will focus on optimizing network designs to reduce resource demands while maintaining or improving performance. Additionally, the current limitation in the availability of image-based air quality datasets restricts this study to single-station forecasts. Expanding the proposed method to larger regions by collecting image data from multiple stations and developing high-accuracy regional forecasting models will be a key focus of future research. These efforts aim to unlock the full potential of visual image data in advancing air quality forecasting on a broader scale.

## Figures and Tables

**Table 1:** Summary of the multi-source dataset.

Data Type	Source	Variables	Resolution	Valid Number
Image data	Shanghai Municipal Bureau of Ecology and Environment, https://sthj.sh.gov.cn/	Surveillance images from a single camera	Hourly, 584 × 389 pixels	8499
Atmospheric data	China National Environmental Monitoring Center, http://www.cnemc.cn/	PM_2.5_, PM_10_, SO_2_, NO_2_, O_3_, CO	Hourly, Site-level	8374
Meteorological data	Copernicus Climate Change Service, https://cds.climate.copernicus.eu/ (accessed on 6 September 2022)	Precipitation, temperature, surface pressure, evaporation relative humidity, wind speed, wind direction,	Hourly, 0.25° × 0.25°	8760
Temporal data	Metadata from datasets	Month, hour	Hourly	8760

**Table 2:** Performance comparison of different methods.

Method	PM_2.5_		PM_10_	
	*R* ^2^	RMSE (µg/m^3^)	*R* ^2^	RMSE (µg/m^3^)
MLR	0.5863	13.26	0.4776	26.91
RF	0.7540	10.22	0.6193	22.97
SVR	0.7055	11.18	0.5862	23.95
LSTM	0.8990	6.55	0.8381	14.98
Ours	0.9459	4.79	0.9045	11.51

**Table 3:** Predictions of pretrain–finetuned and retrained models at Renwu and Linyuan stations.

Pollutants	Metrics	Renwu Station		Linyuan Station	
		Retrain	Pretrain–Finetune	Retrain	Pretrain–Finetune
PM_2.5_	*R* ^2^	0.9102	0.9739	0.9208	0.9717
	RMSE (µg/m^3^)	3.37	1.81	3.29	1.96
PM_10_	*R* ^2^	0.8912	0.9384	0.8914	0.9346
	RMSE (µg/m^3^)	7.19	5.41	7.68	5.96

## Data Availability

The original contributions presented in the study are included in the article; further inquiries can be directed to the corresponding authors.
